# Solute Carrier Family 30 Member 8 Gene 807C/T Polymorphism and Type 2 Diabetes Mellitus in the Chinese Population: A Meta-Analysis Including 6,942 Subjects

**DOI:** 10.3389/fendo.2018.00263

**Published:** 2018-05-23

**Authors:** Yan-Yan Li, Xin-Zheng Lu, Hui Wang, Xin-Xing Yang, Hong-Yu Geng, Ge Gong, Yi-Yang Zhan, Hyun Jun Kim, Zhi-Jian Yang

**Affiliations:** ^1^Institute of Clinical Medicine, First Affiliated Hospital of Nanjing Medical University, Nanjing, China; ^2^Department of Gerontology, First Affiliated Hospital of Nanjing Medical University, Nanjing, China; ^3^Department of Cardiology, First Affiliated Hospital of Nanjing Medical University, Nanjing, China; ^4^Department of Intensive Care Unit, Baoding First Center Hospital, Baoding, China; ^5^Department of Gerontology, Nanjing General Hospital of Nanjing Military Command, Nanjing, China; ^6^Department of Physiology, University of Cincinnati, Cincinnati, OH, United States

**Keywords:** *solute carrier family 30 (zinc transporter) member 8*, 807C/T, polymorphism, Chinese, type 2 diabetes mellitus

## Abstract

**Background:**

Although *solute carrier family 30 (zinc transporter) member 8 (SLC30A8)* gene 807C/T polymorphism is associated with an increased risk of type 2 diabetes mellitus (T2DM) risk, there remains some inconsistency between individual studies.

**Objective:**

The aim of the study is to explore the relationship between *SLC30A8* gene 807C/T polymorphism and T2DM in the Chinese population.

**Methods:**

The current meta-analysis compiles and analyzes the data of 6,942 participants from 10 independent studies. Either a fixed or random-effects model was adopted to evaluate the pooled odds ratio (ORs) and the corresponding 95% confidence interval (95% CI).

**Results:**

A significant association between *SLC30A8* gene 807C/T polymorphism and T2DM was found in the Chinese population under allelic (OR: 0.85, 95% CI: 0.80–0.91, *P* = 7.42 × 10^−7^), recessive (OR: 0.52, 95% CI: 0.38–0.72, *P* = 8.49 × 10^−5^), dominant (OR: 2.40, 95% CI: 1.68–3.41, *P* = 1.30 × 10^−6^), homozygous (OR: 0.52, 95% CI: 0.40–0.67, *P* = 2.90 × 10^−7^), heterozygous (OR: 0.79, 95% CI: 0.71–0.88, *P* = 1.63 × 10^−5^), and additive genetic models (OR: 0.73, 95% CI: 0.64–0.83, *P* = 7.05 × 10^−7^).

**Conclusion:**

*SLC30A8* gene 807C/T polymorphism was significantly associated with an increased T2DM risk in the Chinese population. Therefore, individuals of Chinese descent with the C allele of *SLC30A8* gene 807C/T polymorphism may be more susceptible to developing T2DM, while individuals with the T allele may be protected against T2DM.

## Introduction

The latest data from International Diabetes Federation (IDF) that 1 in 11 adults has diabetes worldwide (total up to 4.15 hundred million). In addition, there are many prediabetic patients. Morbidity associated with prediabetes and diabetes is as high as 6.7 and 8.8%, respectively. If current trends persist, the IDF forecasts that by 2040, 10% of world population will have diabetes with an additional 7.5% of the population categorized as prediabetic.

The prevalence and the complexity of this disease process require further research into these conditions. Type 2 diabetes mellitus (T2DM), also called adult-onset diabetes, usually occurs between the ages of 35–40 and accounts for the vast majority of diabetic disease. Alongside behavioral and environmental factors, genetic factors play a significant role in the pathogenesis of T2DM. The gene of interest in this study is the *solute carrier family 30 member 8 (SLC30A8)* gene. This gene encodes for the zinc transporter-8 (ZnT-8) protein which is embedded into the vesicular membrane of pancreatic β-cells. These vesicles store insulin granular. ZnT-8 could facilitate the binding of zinc and insulin in the cytoplasm of the β-cells and play an important role in the synthesis, maturation, storage, and secretion of insulin.

The *SLC30A8* gene, located on the chromosome 8q24.11, spans about 37 kb and contains eight exons encoding 369 amino acids. The *SLC30A8* gene 807C/T polymorphism (rs13266634) involves the substitution of the cytosine (C) in the 807th position with thymine (T). This changes the arginine (R) in the 325th position to tryptophan (W) (1). This 807C allele would make the protein function change and influence the post-transcription mechanism (2). This mutation result in abnormal Zn^+^ accumulation and reduce the insulin secretion. In 2006, Chimienti et al. found that increased expression of the *SLC30A8* gene increased insulin secretion in response to high plasma glucose (3). Hence, *SLC30A8* gene could play a role in the pathogenesis of T2DM by influencing the pancreatic islet β-cells function.

Although many studies have been performed on the association of *SLC30A8* gene 807C/T polymorphism and increased T2DM risk, the individual studies fail to present a consistent result. In 2016, Su et al. found that *SLC30A8* gene 807C/T polymorphism was significantly associated with T2DM risk and the C allele was the risk allele in a Xinjiang T2DM population (4). Analogously, in 2011, Wang et al. also found that the *SLC30A8* gene rs13266634 polymorphism was associated with T2DM risk and the C allele is the T2DM susceptible allele in a Hunan population (5). On the other hand, Zhong et al. found that there was no significant association between *SLC30A8* gene 807C/T polymorphism and T2DM in another Xinjiang population (6).

In the present meta-analysis, the data from 3,458 T2DM patients and 3,484 was compiled to give insight on the relationship between *SLC30A8* gene 807C/T polymorphism and T2DM in the Chinese population (Table S1 in Supplementary Material).

## Materials and Methods

### Publication Search and Inclusion Criteria

For the initial search, we used the keywords “solute carrier family 30 (zinc transporter) member 8,” “*SLC30A8,”* “*SLC30A8*, 807C/T, Chinese and type 2 diabetes mellitus,” “rs13266634, Chinese and type 2 diabetes mellitus” in the following databases: PubMed, the Wanfang database, the VIP database, and the China National Knowledge Infrastructure. The 20 studies initially pulled from this search, were published 2008–2016 with the last research updated on January 11, 2018.

The inclusion criteria of the studies were as follows: (a) evaluation of the relationship of *SLC30A8* gene 807C/T polymorphism and T2DM in the Chinese population; (b) T2DM diagnosis based on criteria proposed by World Health Organization in 1999: 2 h plasma glucose of the oral glucose tolerance test ≥11.1 mmol/L or fasting plasma glucose ≥7.0 mmol/L. (c) Officially published cohort or case–control studies. (d) The *SLC30A8* gene 807C/T polymorphism in the control group conforms to Hardy–Weinberg equilibrium (HWE).

### Data Extraction

The information was extracted out on the basis of a standard protocol. Three investigators were in charge of gathering the data from the retrieved studies. Two independently searched for duplicate studies and a third was responsible for resolving any disagreements. Duplicate studies and those that either deviated from the inclusion criteria or could not supply sufficient data were removed from the current meta-analysis. Similar datasets presented in different publications by the similar author group were incorporated into the analysis only once. The extracted data, including the first author’s name, publication year, region, genotyping method, matching criteria, genotype number, and total number of cases and controls, are displayed in the Table 1.

**Table 1:** Characteristics of the investigated studies of the association between *SLC30A8* gene 807C/T (rs13266634 C/T) polymorphism and T2DM in the Chinese population.

Author	Year	Region	T2DM			Control			Genotype method	Matching criteria	Sample size (T2DM/control)
							
			CC	CT	TT	CC	CT	TT			
Wang et al. (14)	2008	Chongqing	152	219	83	87	141	83	PCR-RFLP	Sex, DBP, ethnicity	454/311
Wang et al. (5)	2011	Hunan	82	117	37	48	95	75	PCR-RFLP	Sex, HDL-C, ethnicity	120/236
Yu et al. (15)	2013	Hubei	33	46	18	70	133	98	Real-time fluorescence PCR	FPG, ethnicity	97/301
Liu et al. (16)	2015	Liaoning	52	64	20	32	84	29	PCR-HRM	Age, sex, ethnicity	136/145
Zhang et al. (17)	2014	Gansu	48	56	19	30	65	30	PCR-RFLP	Age, sex, ethnicity	123/129
Li et al. (18)	2011	Neimenggu	36	81	8	24	55	18	AS-PCR	Age, sex, BMI, ethnicity	125/97
Su et al. (4)	2016	Xinjiang	466	402	64	432	403	97	Multiplex PCR	Age, sex, BMI, DBP, TG, ethnicity	932/932
Zhong and Chang (6)	2013	Xinjiang	23	46	31	27	50	23	PCR-RFLP	Age, sex, HDL-C, DBP, ethnicity	100/100
Zhang et al. (19)	2015	Gansu	107	116	40	66	141	55	PCR-RFLP	Age, sex, SBP, DBP, ethnicity	263/262
Han et al. (20)	2010	Beijing	386	457	149	327	487	179	Real-time fluorescence PCR	BMI, ethnicity	992/993

*T2DM, type 2 diabetes mellitus; FPG, fasting plasma glucose; DBP, diastolic blood pressure; PCR-HRM, polymerase chain reaction-high resolution melting; TG, triglyceride; TC, total cholesterol; BMI, body mass index; PCR-RFLP, polymerase chain reaction-restriction fragment length polymorphism; AS-PCR, allele-specific PCR*.

### Statistical Analysis

Six genetic models were used in this analysis: allelic (T allele distribution frequency of *SLC30A8* gene 807C/T polymorphism), recessive (TT vs. CT + CC), dominant (CC vs. TT + TC), homozygous (TT vs. CC), heterozygous (CT vs. CC), and additive (total T vs. total C). We used odds ratio (OR) and its corresponding to its 95% confidence interval (CI) to compare the association of *SLC30A8* gene 807C/T polymorphism and T2DM. But the model we would use to calculate the OR is dependent on the heterogeneity of the dataset. We calculated the heterogeneity between the individual studies by using the Chi-square-based *Q*-test with the significance level set at *P* < 0.05 (7). If heterogeneity was detected, the fixed-effects model, or the Mantel–Haenszel method, but if heterogeneity was not detected we would use the random-effect model, or DerSimonian and Laird method (8, 9). *Z*-test was used to calculate the pooled OR with the significance set at *P* < 0.05 level. Fisher’s exact test was used to evaluate the HWE with the significance set at *P* < 0.05 level. We looked for potential publication bias by using a funnel plot and assessed for asymmetry using Egger’s linear regression test (*P* < 0.05) (10). Revman 5.0 and STATA 12.0 software (StataCorp, College Station, TX, USA) was used to conduct the statistical analyses in this meta-analysis.

## Results

### Studies and Populations

Of the 20 papers pulled from the initial search, 10 papers met our inclusion criteria. Among the 10 excluded studies, three studies deviated from the HWE (11–13). Four studies were of review character and another four studies were irrelevant to either T2DM or the *SLC30A8* gene 807C/T gene polymorphism. The total data were extracted from 3,458 T2DM patients and 3,484 controls (Table 1; Table S2 in Supplementary Material) (4–6, 14–20). Population from the studies was from eight different Chinese provinces: Beijing, Chongqing, Hunan, Hubei, Liaoning, Gansu, Neimenggu, and Xinjiang.

### Pooled Analyses

**Figure 1:**
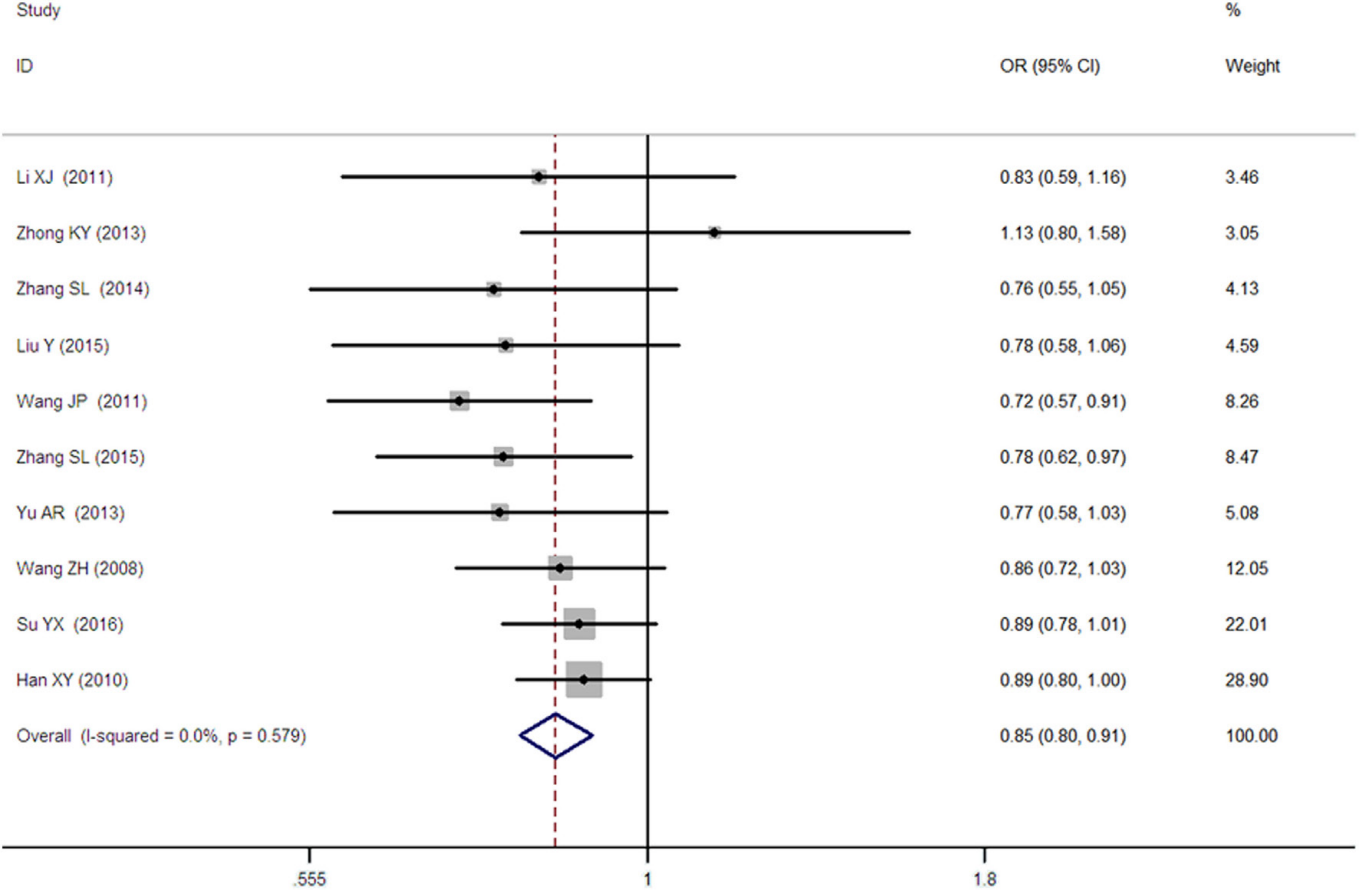
Forest plot of type 2 diabetes mellitus associated with *SLC30A8* gene rs13266634C/T polymorphism in the Chinese population under an allelic genetic model (distribution of T allele frequency of *SLC30A8* gene).

**Figure 2:**
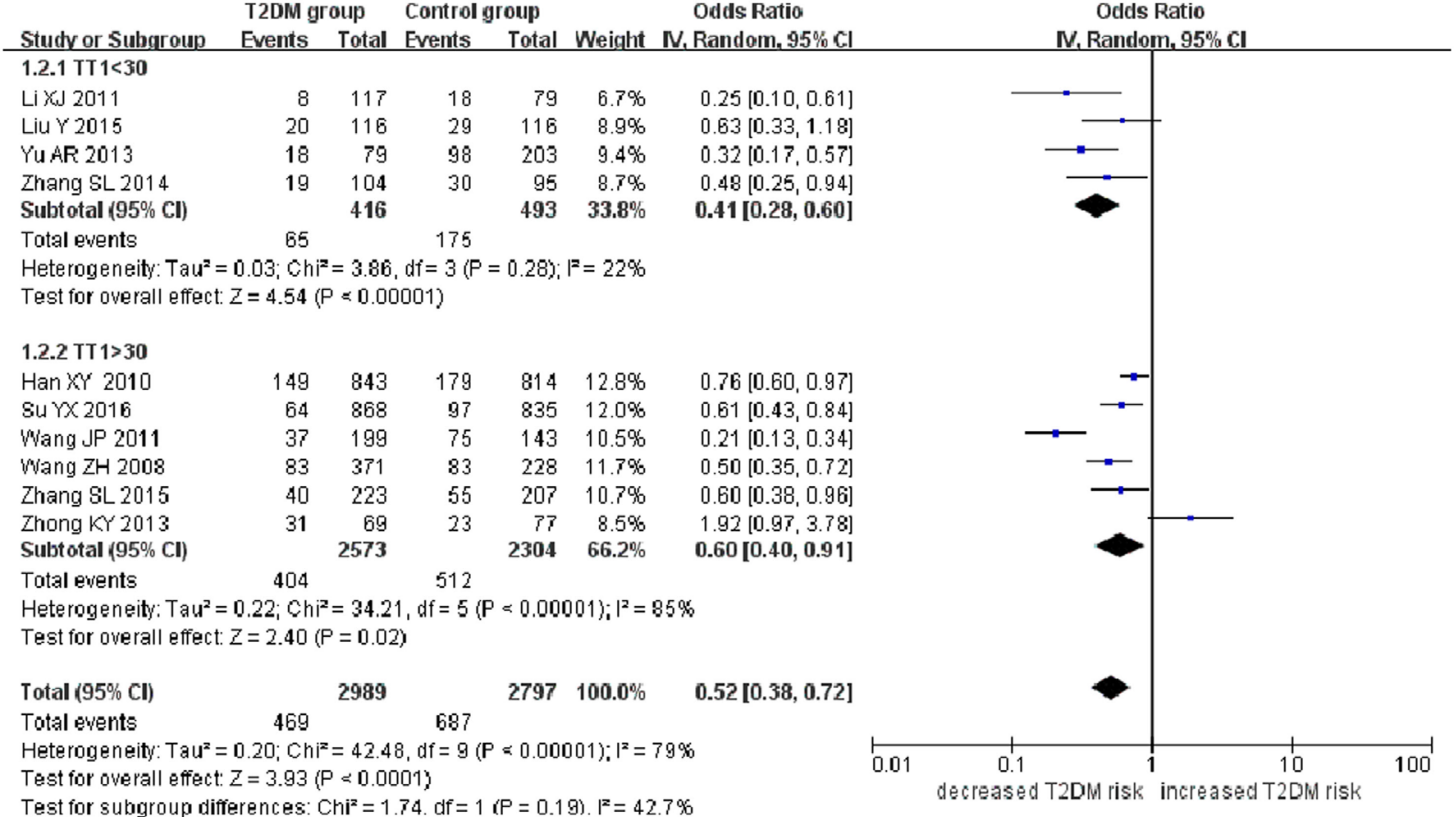
Forest plot of type 2 diabetes mellitus associated with *SLC30A8* gene rs13266634C/T polymorphism in the Chinese population under a recessive genetic model (TT vs. CT + CC).

**Figure 3:**
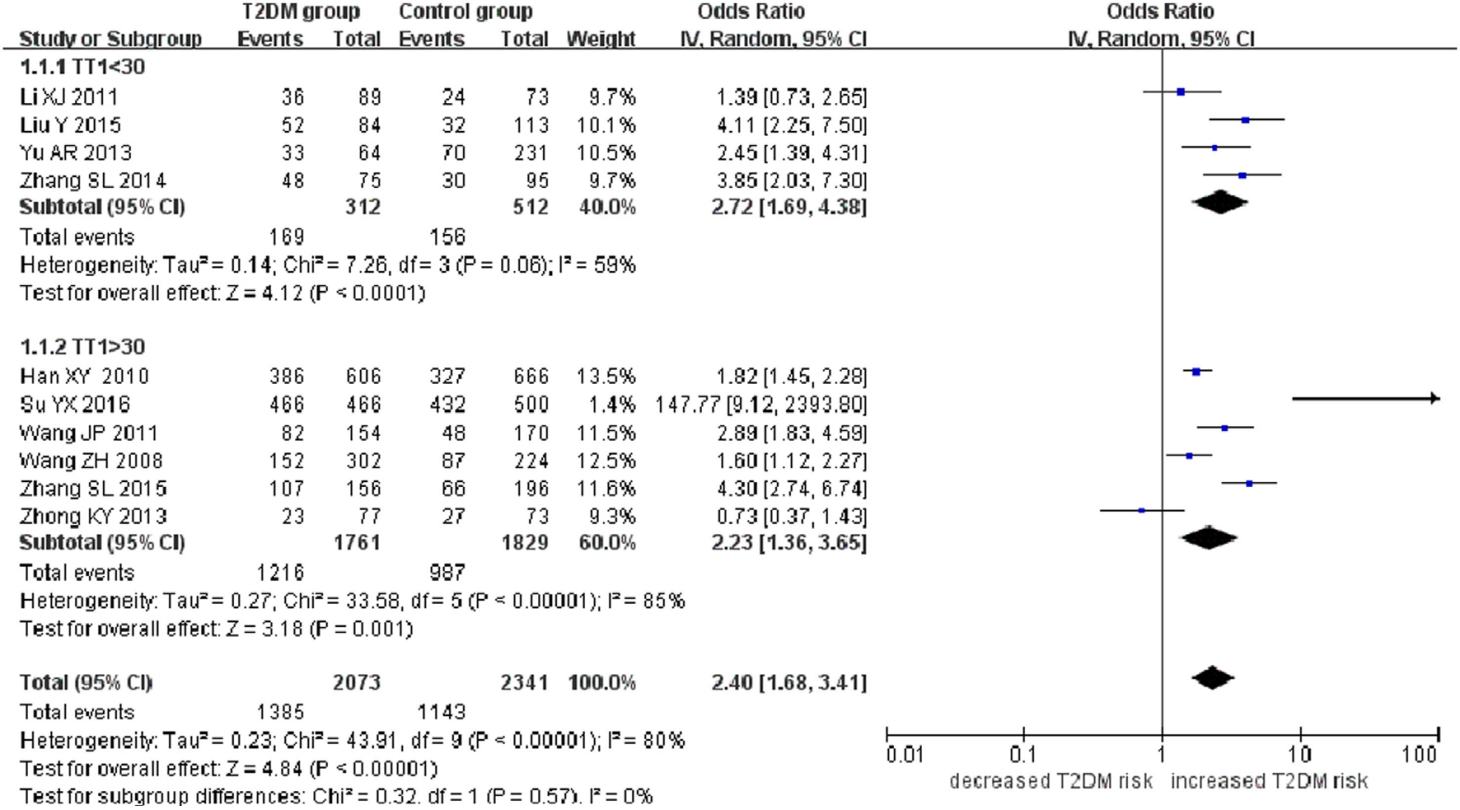
Forest plot of type 2 diabetes mellitus associated with *SLC30A8* gene rs13266634C/T polymorphism in the Chinese population under a dominant genetic model (CC vs. CT + TT).

**Figure 4:**
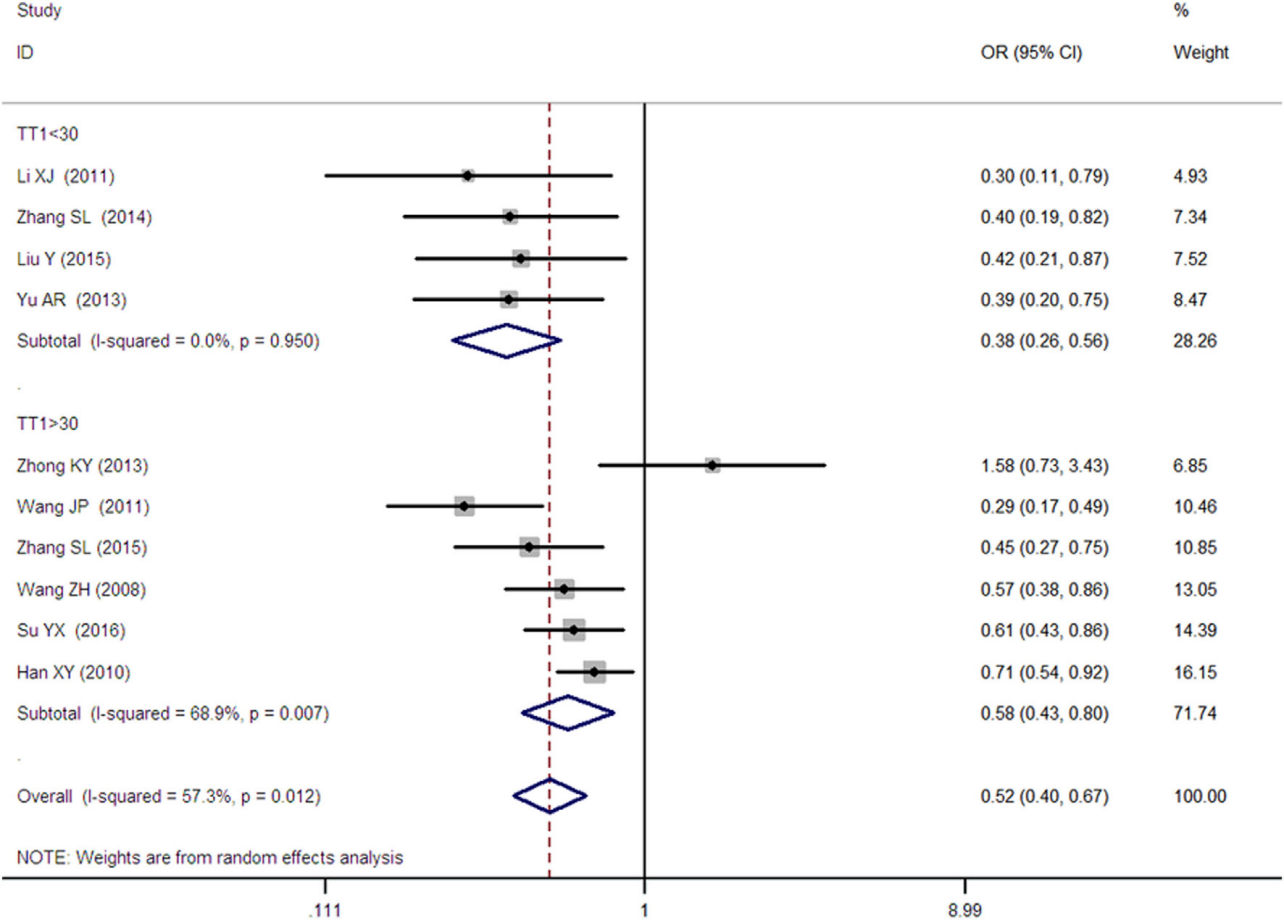
Forest plot of type 2 diabetes mellitus associated with *SLC30A8* gene rs13266634C/T in the Chinese population under a homozygous genetic model (TT vs. CC).

**Figure 5:**
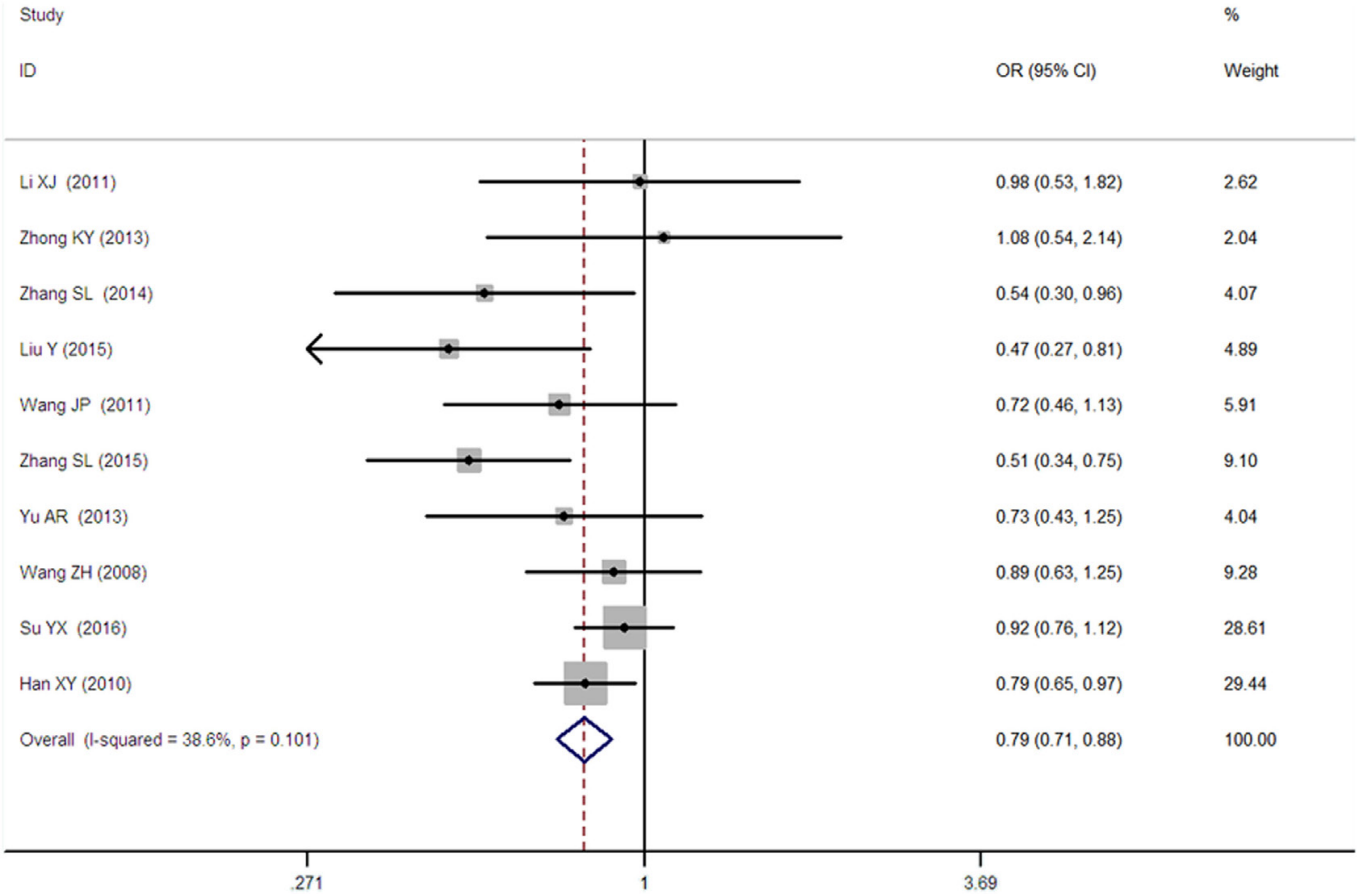
Forest plot of type 2 diabetes mellitus associated with *SLC30A8* gene rs13266634C/T in the Chinese population under a heterozygous genetic model (CT vs. CC).

**Figure 6:**
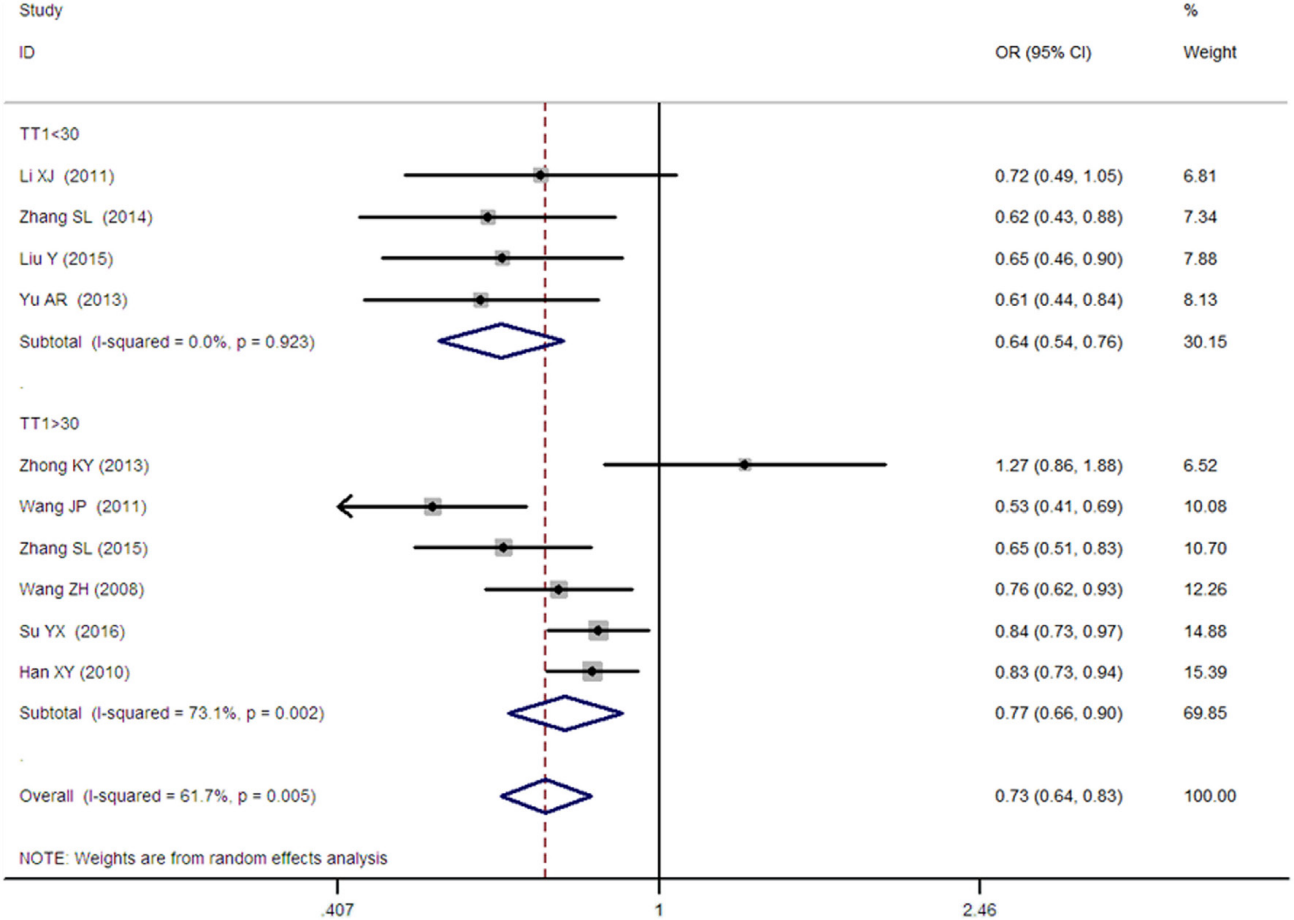
Forest plot of type 2 diabetes mellitus associated with *SLC30A8* gene rs13266634C/T in the Chinese population under an additive genetic model (T vs. C).

A significant association between *SLC30A8* gene 807C/T polymorphism and T2DM was found in the Chinese population under allelic (OR: 0.85, 95% CI: 0.80–0.91, *P* = 7.42 × 10^−7^), recessive (OR: 0.52, 95% CI: 0.38–0.72, *P* = 8.49 × 10^−5^), dominant (OR: 2.40, 95% CI: 1.68–3.41, *P* = 1.30 × 10^−6^), homozygous (OR: 0.52, 95% CI: 0.40–0.67, *P* = 2.90 × 10^−7^), heterozygous (OR: 0.79, 95% CI: 0.71–0.88, *P* = 1.63 × 10^−5^), and additive genetic models (OR: 0.73, 95% CI: 0.64–0.83, *P* = 7.05 × 10^−7^) (Figures 1–6; Table 2).

**Table 2:** Summary of meta-analysis of association between *SLC30A8* gene 807C/T (rs13266634C/T) polymorphism and T2DM in the Chinese population.

Genetic model	Pooled OR (95% CI)	*Z* value	*P* value	Literature number	T2DM size	Control size	*P*_heterogeneity (_*_I_*^2^%_)_
Allele genetic model	0.85 (0.80–0. 91)	4.95	7.42 × 10^−7^*	10	3,458	3,484	0.579 (0%)
Recessive genetic model	0.52 (0.38–0.72)	3.93	8.49 × 10^−5*^	10	3,458	3,484	<0.00001* (79.0%)
TT1 < 30	0.41 (0.28–0.60)	4.54	5.63 × 10^−6*^	4	484	467	0.28 (22.0%)
TT1 > 30	0.60 (0.40–0.91)	2.40	0.02*	6	2,974	3,017	<0.00001* (85.0%)
Dominant genetic model	2.40 (1.68–3.41)	4.84	1.30 × 10^−6*^	10	3,458	3,484	<0.00001* (80.0%)
TT1 < 30	2.72 (1.69–4.38)	4.12	3.79 × 10^−5*^	4	484	467	0.06 (59.0%)
TT1 > 30	2. 23 (1.36–3.65)	3.18	0.001*	6	2,974	3,017	<0.00001* (85.0%)
Homozygous genetic model	0.52 (0.40–0.67)	5.13	2. 90 × 10^−7^*	10	3,458	3,484	0.012* (57.3%)
TT1 < 30	0.38 (0.26–0.56)	5.02	5.17 × 10^−7^*	4	484	467	0.95 (0%)
TT1 > 30	0.58 (0.43–0.80)	3.35	0.001*	6	2,974	3,017	0.007* (68.9%)
Heterozygous genetic model	0.79 (0.71–0.88)	4.31	1.63 × 10^−5*^	10	3,458	3,484	0.101* (38.6%)
Additive genetic model	0.73 (0.64–0.83)	4.96	7.05 × 10^−7^*	10	3,458	3,484	0.005* (61.7%)
TT1 < 30	0.64 (0.54–0.76)	4.99	6.04 × 10^−7^*	4	484	467	0.923 (0%)
TT1 > 30	0.73 (0.64–0.83)	3.22	0.001*	6	2,974	3,017	0.002* (73.1%)

*P < 0.05.

*SLC30A8, solute carrier family 30 (zinc transporter) member8; T2DM, type 2 diabetes mellitus; CI, confidence interval; OR, odds ratio; T2DM size, the total number of T2DM cases; control size, the total number of control group; allele genetic model, T allele distribution frequency; recessive genetic model, TT vs. CC + CT; dominant genetic model, CC vs. CT + TT; homozygous genetic model, TT vs. CC; heterozygous genetic model, CT vs. CC; additive genetic model, total T allele vs. total C*.

As heterogeneity was detected under the recessive, dominant, homozygous, and additive genetic models (recessive *P*_heterogeneity_ < 0.00001, *I*^2^ = 79.0%; dominant *P*_heterogeneity_ < 0.00001, *I*^2^ = 80.0%; homozygous *P*_heterogeneity_ = 0.012, *I*^2^ = 57.3%; and additive *P*_heterogeneity_ = 0.005, *I*^2^ = 61.7%), we performed a meta-regression to explore the source of heterogeneity and found the TT genotype number in the T2DM group (TT1) was determined to be the main source of heterogeneity source. The subgroup analysis stratified by TT1 has been conducted under these genetic models. When analyzed by subgroups of TT1 <30 and TT1 >30 subgroup, we reproduced our findings recessive, dominant, homozygous, and additive genetic models (*P* < 0.05), while reducing heterogeneity in the TT1 <30 subgroups (*P*_heterogeneity_ < 0.05) while retaining heterogeneity in the TT1 >30 subgroups under aforementioned genetic models (*P*_heterogeneity_ > 0.05) (Figures 1–6; Table 2).

### Bias Diagnostics

**Figure 7:**
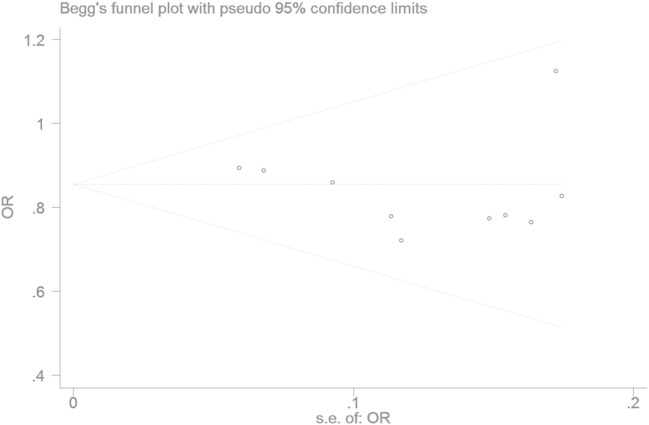
Begger’s funnel plot for studies of type 2 diabetes mellitus associated with *SLC30A8* gene rs13266634C/T in the Chinese population under a recessive genetic model (TT vs. CT + CC). The horizontal and vertical axis correspond to the OR and confidence limits. Abbreviation: OR, odds ratio.

**Figure 8:**
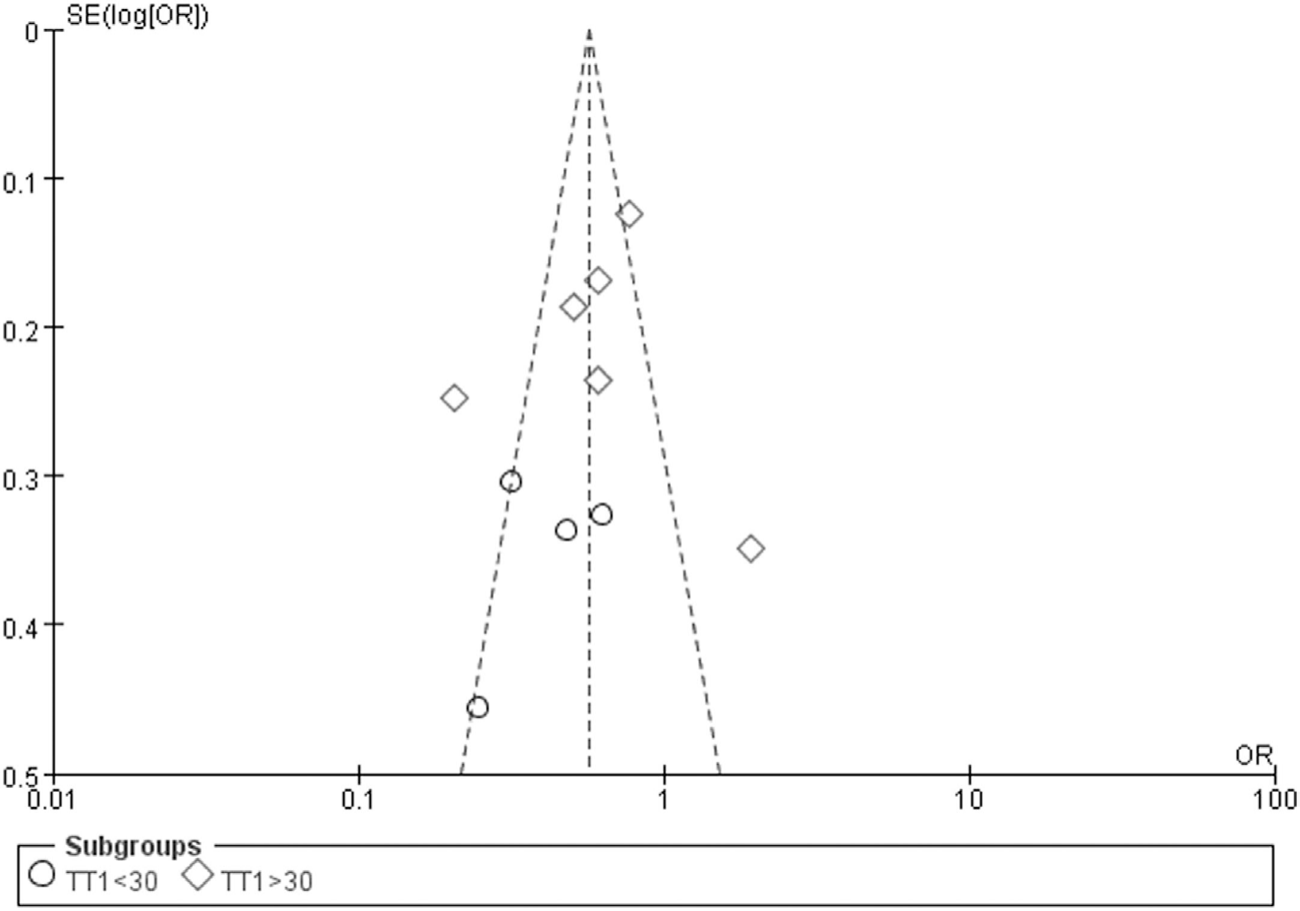
Funnel plot for studies of type 2 diabetes mellitus associated with *SLC30A8* gene rs13266634C/T in the Chinese population under a recessive genetic model (TT vs. CT + CC). The horizontal and vertical axis correspond to the OR and confidence limits. Abbreviation: OR, odds ratio.

The Egger’s test result has shown that there was no significant publication bias under the allele genetic model in this meta-analysis (*T* = −0.93, *P* = 0.379) (Figure 7). Moreover, no publication bias was visualized in the funnel plot under the recessive genetic model (Figure 8).

## Discussion

In this meta-analysis, a significant association was observed between *SLC30A8* gene 807C/T polymorphism and T2DM in the Chinese population under the allele (OR: 0.85), recessive (OR: 0.52), dominant (OR: 2.40), homozygous (OR: 0.52), heterozygous (OR: 0.79), and additive (OR: 0.73) genetic models. Hence, in the present meta-analysis we concluded that Chinese carriers of the C allele of the *SLC30A8* gene 807C/T polymorphism may be at an increased risk of developing T2DM.

Since the heterogeneity was detected under recessive, dominant, homozygous, and additive genetic models, the random genetic model was used. Furthermore, meta-regression was subsequently performed to explore the heterogeneity source. TT1 was the main heterogeneity source under the recessive, dominant, homozygous, and additive genetic models. The heterogeneity was significantly reduced in the subgroup analysis stratified by TT1 which suggested that TT1 was indeed the main heterogeneity source under these genetic models. In addition, as no heterogeneity was detected under the allele and heterozygous genetic models, the fixed genetic model was used.

Although insulin resistance of peripheral tissue was major investigative target of early research into T2DM, researchers subsequently found that defective secretion of insulin is required for normal glucose tolerance to progress toward hyperglycemia (19). This is reinforced by prospective observations that pancreatic beta cells of newly diagnosed diabetic patients have half the functionality of a normal healthy individual with a predicted decline of 4.5% annually. If we were to follow the curve backwards, however, researchers estimate that these patients had 100% of beta cell functioning 12 years before diagnosis (21).

In 2010, Li et al. explored the relationship between *SLC30A8* gene rs13266634C/T polymorphism and T2DM in a Hengyang population and found significantly decreased scores on the homeostasis model assessment-β in patients with the TT, CT, and CC genotype (22). This suggested a potential link between the 807C allele and defective insulin secretion. Cohort studies of other non-Chinese populations also corroborated his result. Boesgaard et al. found that among non-diabetic (NDM) descendants of T2DM population, patients with the 807C allele had a reduced acute insulin response (AIR) during an intravenous glucose tolerance test (23). More specifically, AIR in CC genotype patients was 19% lower than that of TT genotype patients. This demonstrated the impaired pancreatic islet β cell function in patients with the 807C allele even though they were not yet diagnosed with T2DM. In 2008, Cauchi et al. analyzed novel risk loci for T2DM in a general French population with normal blood glucose level and found that the fasting insulin level in the rs13266634 C allele carriers already significantly decreased than that in the non rs13266634 C allele carriers at baseline (24). Wang et al. found that the insulin sensitivity in rs13266634 C allele carriers is higher than that in the non rs13266634 C allele carriers in the NGT persons. It is also the reason for they could maintain the normal tolerance with the low serum insulin level (14).

The mechanism underlying the relationship between *SLC30A8* gene rs13266634C/T polymorphism, T2DM, and pancreas islet β cells dysfunction is likely associated with its molecular action. The ZnT-8 protein encoded by *SLC30A8* gene is located in insulin-containing vesicle membrane in pancreatic islet β cells and is implicated in the zinc transport into the vesicle (25). Insulin then binds with two Zn^2+^ ions to form a stable hexameric structure. The insulin will only be secreted out when the pancreas islet β cells are stimulated (26). Hence, SLC30A8 is the dominant sector participating the insulin synthesis, storage, and secretion. When released, Zn may also inhibit the neighboring pancreas islet α cells from releasing glucagon and attenuate the pancreas islet β cells apoptosis (27, 28). The *SLC30A8* gene rs13266634 C allele would lead to the abnormal ZnT-8 structure and function, and cause decreased insulin secretion and increased glucagon secretion, pancreas islet β cells apoptosis, blood glucose levels. The current meta-analysis results confirmed this conclusion. In 2007, Zeggini et al. performed a genome-wide association study on the *SLC30A8* gene rs13266634C/T polymorphism and T2DM. They genotyped rs13266634 independently and obtained replication of this finding in the French scan (29).

In 2003, Chen et al. have performed a meta-analysis on the association of *SLC30A8* gene rs13266634C/T polymorphism and T2DM (30). In 2015, Cheng et al. also conducted this meta-analysis and replicated the same conclusion (31). In 2016, Fan et al. also reported that *SLC30A8* gene polymorphism was associated with T2DM increased risk in African and European populations as well as Asian groups (32). However, in all of the above-mentioned meta-analyses, the authors ignored the ethnicity difference and mixed the Chinese population with other populations to analyze the association. Furthermore, the study by Huang (13) deviating from the HWE was still included in Fan’s meta-analysis which was not consistent with their exclusion criteria. Hence, their results may not be as objective or credible as the current meta-analysis.

The large-scale studies on the association of T2DM and *SLC30A8* gene rs13266634C/T polymorphism are still needed to further explore this connection. As T2DM is a disease of multifactorial inheritance and other gene polymorphisms as *SUMO4* gene M55V polymorphism (33), *LEPR* gene Gln223Arg polymorphism (34), and *TCF7L2* gene rs12255372G/T polymorphism (35) are also associated with T2DM susceptibility. The potential effects of these polymorphisms, independently and synergistically, were not incorporated into the analysis.

In conclusion, our meta-analysis found a significant association between *SLC30A8* gene rs13266634C/T polymorphism and an increased risk of T2DM in the Chinese population. Although large-scale studies are still required to test our result, this finding may present a small step toward developing a personalized therapy to T2DM in the Chinese population.

## Author Contributions

Conceived and designed the experiments: Y-YL. Performed the experiments: Y-YL, GG, H-YG, and HW. Analyzed the data: Y-YL, X-XY, and X-ZL. Contributed reagents/material/analysis tools: Y-YL and HW. Wrote the manuscript: Y-YL and HK. Reference collection and data management: Y-YL and X-ZL. Statistical analyses and paper writing: Y-YL. Study design: Y-YL, Y-YZ, and Z-JY.

## Conflict of Interest Statement

The authors report no relationships that could be construed as a conflict of interest.
