# Supplementary material for: Solute Carrier Family 30 Member 8 Gene 807C/T Polymorphism and Type 2 Diabetes Mellitus in the Chinese Population: A Meta-Analysis Including 6,942 Subjects
Source: Front Endocrinol (Lausanne). 2018 May 23;9:263. doi: 10.3389/fendo.2018.00263 (PMC5974095; doi:10.3389/fendo.2018.00263)
Supplement: Supplementary file 2 [file table_2.DOCX]

**PRISMA 2009 Flow Diagram**


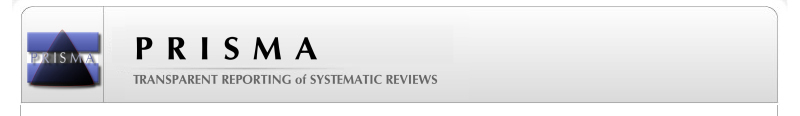


Records removed for repeated publication
(n = 0 )

Records rejected for no association with T2DM or *SLC30A8* gene 807C/T gene polymorphism

(n =4)

Full-text articles removed for deviation from HWE (n =2)

Records rejected for review characteristic
(n =4 )

Studies included in the meta-analysis
(n =9)

Full-text articles evaluated for eligibility
(n =13)

Records screened
(n =15 )

Records after duplicates excluded
(n =19)

Additional records retrived through other sources
(n =0 )

## Identification

## Eligibility

## Included

## Screening

Records retrieved through database searching
(n =19 )
